# Inhibitory Effects of Rhaponticin on Osteoclast Formation and Resorption by Targeting RANKL-Induced NFATc1 and ROS Activity

**DOI:** 10.3389/fphar.2021.645140

**Published:** 2021-09-23

**Authors:** Jianbo He, Kai Chen, Tiancheng Deng, Jiewei Xie, Kunjing Zhong, Jinbo Yuan, Ziyi Wang, Zhifeng Xiao, Ronghe Gu, Delong Chen, Xiaojuan Li, Dingkun Lin, Jiake Xu

**Affiliations:** ^1^ The Second Affiliated Hospital of Guangzhou University of Chinese Medicine, Guangdong Provincial Hospital of Chinese Medicine, Guangzhou, China; ^2^ School of Biomedical Sciences, University of Western Australia, Perth, WA, Australia; ^3^ Department of Orthopedics, First People’s Hospital of Nanning, Fifth Affiliated Hospital of Guangxi Medical University, Nanning, China; ^4^ The First Affiliated Hospital of Guangzhou University of Chinese Medicine, Guangzhou, China; ^5^ Formula-Pattern Research Center, School of Traditional Chinese Medicine, Jinan University, Guangzhou, China

**Keywords:** osteoclast, NFATc1, ROS, rhaponticin, bone

## Abstract

The extravagant osteoclast formation and resorption is the main cause of osteoporosis. Inhibiting the hyperactive osteoclastic resorption is considered as an efficient treatment for osteoporosis. Rhaponticin (RH) is a small molecule that has been reported to possess anti-inflammatory, anti-allergic, anti-fibrotic, and anti-diabetic activities. However, the influence of RH on osteoclasts differentiation and function is still unclear. To this end, an array of assays including receptor activator of nuclear factor kappa-Β (NF-κB) ligand (RANKL) induced osteoclastogenesis, tartrate-resistant acidic phosphatase (TRAcP) staining, immunofluorescence, and hydroxyapatite resorption were performed in this study. It was found that RH had significant anti-catabolic effects by inhibiting osteoclastogenesis and bone resorption without cytotoxicity. Mechanistically, the expression of NADPH oxidase 1 (Nox1) was found to be suppressed and antioxidant enzymes including catalase, superoxide dismutase 2 (SOD-2), and heme oxygenase-1(HO-1) were enhanced following RH treatment, suggesting RH exhibited antioxidant activity by reducing the generation of reactive oxygen species (ROS) as well as enhancing the depletion of ROS. In addition, MAPKs, NF-κB, and intracellular Ca^2+^ oscillation pathways were significantly inhibited by RH. These changes led to the deactivation of osteoclast master transcriptional factor-nuclear factor of activated T cells 1 (NFATc1), as examined by qPCR and Western blot assay, which led to the decreased expression of downstream integrin β3, c-Fos, cathepsin K, and Atp6v0d2. These results suggested that RH could effectively suppress RANKL-regulated osteoclast formation and bone resorption. Therefore, we propose that RH can represent a novel natural small molecule for the treatment of osteoporosis by inhibiting excessive osteoclast activity.

## Introduction

The well-orchestrated bone formation and bone resorption is vital for bone modelling and remodelling (Baron & Kneissel, 2013). Excessive bone resorption is considered to be the main cause of osteoporosis which is characterized by reduced bone mass, with the high risk of bone fractures (Rachner, Khosla, & Hofbauer, 2011). Estrogen deficiency and glucocorticoid administration are the top two primary causes of osteoporosis (Adami & Saag, 2019; Manolagas, O'Brien, & Almeida, 2013) and subsequent vertebral or hip fractures pose heavy economic burdens to patients and society (Melton, 2003). Clinically available osteoporosis treatments such as hormone replacement therapy, bisphosphates, and RANKL antibody have acceptable therapeutic outcome by exhibiting an appreciated suppression on bone resorption. However, a series of potential adverse impacts including breast cancer, endometrial carcinoma, heart attack, and jaw osteonecrosis will have some limitation on their application (Ross, Paganini-Hill, Wan, & Pike, 2000; Lopez-Jornet, Camacho-Alonso, Molina-Minano, & Gomez-Garcia, 2010; Wang et al., 2017). Therefore, it is urgent to provide more effective alternative options to alleviate these osteoclast-related bone disorders.

Osteoclasts (OCs) are bone-resorbing and multinucleated cells, differentiating from the bone marrow monocytes (BMMs) (Ibbotson, Roodman, McManus, & Mundy, 1984). Mature osteoclasts are the unique cell lineage which can resorb bone tissue and thus highly involved in the bone metabolism. During the process of differentiation, there are two indispensable factors: macrophage colony-stimulating factor (M-CSF) and RANKL (Teitelbaum, 2000). M-CSF maintains the proliferation and survival of osteoclast precursor BMMs (Teitelbaum, 2000). RANKL mediates the differentiation of osteoclast precursor cells and regulates the function of mature osteoclasts by binding to its receptor–RANK (Wada, Nakashima, Hiroshi, & Penninger, 2006). RANKL-induced intracellular signalling pathways include reactive oxygen species (ROS), NF-κB, mitogen-activated protein kinase (MAPK) and calcium oscillation, which collaboratively induce the differentiation and function of osteoclasts (Wada, et al., 2006). Eventually, two main transcription factors–activator protein 1 (AP-1) and nuclear factor of activated T cells 1 (NFATc1) are activated to enhance osteoclast-specific markers such as tartrate-resistant acid phosphatase (TRAcP) and cathepsin K (CTSK) (Indo et al., 2013; Wada, et al., 2006). Hence, strategies on suppressing the RANKL-induced pathways in osteoclasts are deemed practical for the treatment of osteoporosis.

Rhaponticin (RH) is a natural compound originally isolated from the famous Chinese medicinal herb *Rheum undulatum L.*. It is well-known for anti-inflammatory, anti-allergic, anti-fibrotic, and anti-cancer effects (Kim & Ma, 2018; Tao et al., 2017; Wei et al., 2017). RH suppresses the spread and expansion of cancer cells by decreasing its transmutation and angiogenic functions (Kim & Ma, 2018). RH also effectively prevents pulmonary fibrosis via the modification of AMP activated protein kinase (AMPK) activation and transforming growth factor beta (TGF-β)/Smad pathway *in vitro* and *in vivo* (Tao, et al., 2017). Given the wide range of bioactivities that RH exhibited, we hypothesized RH may affect osteoclast formation and function. In this study, we identified that RH could significantly inhibit RANKL-induced osteoclast formation and resorption by targeting RANKL-induced ROS and NFATc1. The underlying mechanisms include the suppression on MAPK, NF-κB and Ca^2+^ oscillations. Therefore, these results suggested the potential and beneficial anti-catabolic effects of RH on osteoclast-related bone disorders.

## Materials and Methods

### Materials and Reagents

Rhaponticin (purity >98%) was purchased from Ruifensi company (Chengdu, China) and dissolved with dimethyl sulfoxide (DMSO), stocking at the concentration of 100 mM in −20°C freezer. Further dilution was achieved through adding phosphate-buffered saline (PBS) to the original stock. The complete cell medium consisted of Alpha modified minimal essential medium, penicillin/streptomycin (1%), and fetal bovine serum (10%), obtained from Thermo Fisher Scientific (Scoresby, Vic, Australia). Recombinant M-CSF and glutathione S-transferase-recombinant RNAKL (GST-rRANKL) were used as previously described (Xu et al., 2000). The regents for MTS and luciferase assay were purchased from Promega (Madison, WI, United States). The primary antibodies special for NFATc1, c-Fos, CTSK, V-ATPase-d2, integrin β3, extracellular signal regulated kinase (ERK), phosphorylated ERK (*p*-ERK), P38, phosphorylated P38 (p-P38), and β-actin were obtained from Cell Signaling Technology, Santa Cruz Biotechnology and Abcam companies.

### Cell Culture

The BMMs were extracted from the femur and tibia of 10 weeks C57BL/6J mice, and the procedures were in conformity with the principles of the Animal Ethics Committee of the University of Western Australia (RA/3/100/1601). The bone marrow was flushed from the long bones, with the filtering and centrifuge, the bone marrow extracts were cultured in osteoclast cells special medium with the existence of M-CSF at the concentration of 50 ng/ml, and changed the medium every 2 days.

### Cytotoxicity Assay

The BMMs were seeded into the 96-well plate (5 × 10^3^ cells per well) with the M-CSF kit, without the stimulation of RANKL. On the following day, different concentrations of RH were added to the medium for culturing 48 hours. Then the cell viability was determined at the 490 nm absorbance after 2 hours incubation with MTS kit (10 µl/well).

### *In vitro* Osteoclastogenesis Assay

BMMs at the stage of passage 2 were seeded into the 96-well plate (5 × 10^3^ cells per well) and cultured with complete medium containing M-CSF with the overnight to adhere. On the following day, the BMMs were changed to the medium with RANKL (50 ng/ml), in the presence or absence of RH at the varying concentrations (6.25,12.5, 25 and 50 μM). The culturing cell medium needed to be changed about three times every 2 days until the osteoclasts formed after RANKL stimulus. Then the mature cells were fixed with 2.5% glutaraldehyde for 10 mins, with the washing by PBS three times, they were well prepared for TRAcP staining. The cells with more than three nuclei were recognized as the TRAcP-positive osteoclasts, counting under the light microscope.

Furthermore, the time course was undertaken to investigate the effects of RH on definite stages of osteoclastogenesis. The RH was added to the medium with RANKL at an early stage (1–2 days), middle-stage (3–4 days), late-stage (5–6 days) and the whole stage (1–6 days). Finally, the osteoclasts were fixed for TRAcP staining as described above.

### Immunofluorescent Staining

The BMMs were seeded in the 96-well plates with the culturing of M-CSF at the above concentration overnight. Cells were then stimulated by the consistent concentration of RANKL, with the RH treatment (25 and 50 μM, respectively) or not. When the mature osteoclasts formed, 4% paraformaldehyde was added in the wells to fix about 15 minutes at room temperature. After the wash with PBS and permeabilization with 0.25% Triton X-100, the prepared cells were then blocked at the room tempreture for 1 hour with 3% bovine serum albumin. Next, they were probed with the effects of Rhodamine-Phalloidin for the staining of F-actin in the dark. And the nuclei of mature osteoclasts were counterstained with DAPI. Then, they were visualized on the confocal fluorescence microscope (Nikon, A1S confocal microscopy).

### Hydroxyapatite Resorption Assay

The bone resorption could be detected with the hydroxyapatite resorption assay induced by the osteoclasts as described previously (Chen et al., 2019)**.** The primary BMMs were planted into the 6-well collagen-coated plate with the stimulation of M-CSF and RANKL with the above concentration. The mature osteoclast cells were transferred into the 96-well plate with the coat of hydroxyapatite after gently detaching them from the previous plate with special cell dissociation solution (Sigma-Aldrich). With the stimulation of RANKL, the osteoclasts in 96-well hydroxyapatite plate were incubated with the RH at indicated 25 and 50 μM concentrations for 48 hours to fully display the bone resorption function. Then one half of wells were fixed for TRAcP staining for counting the number of multinucleated TRAcP-positive cells in the well, as described above, and the other half wells were flushed with the bleach to remove the adhering cells and measure the resorbed areas on hydroxyapatite surface by osteoclasts.

### RNA Isolation and RT-PCR Analysis

After the maturation of osteoclasts from the primary BMMs by the stimulation of M-CSF and RANKL, with the treatment of RH or not, as mentioned above, the total RNA of different groups was isolated with 1 ml Trizol reagent per well, the procedures were practised according to the manufacturer’s protocol. cDNA was then generated from the RNA samples, with the reagents of M-MLV reverse transcriptase and oligo dT primers. The specific amplification sequences of polymerase chain reaction (PCR) was underdone with the detection of SYBR Green MasterMix, following the special conditions. The related primers were used for detecting gene expression as shown: *Nfatc1* (Forward: 5′-CA ACG​CCC​TGA​CCA​CCG​ATA​G-3′; Reverse: 5′-GGC​TGC​CTT​CCG​TCT​CAT​AGT- 3′), *Atp6v0d2* (Forward: 5′-GTG​AGA​CCT​TGG​AAG​ACC​TGA​A-3′; Reverse: 5′-GAG​AAA​TGT​GCT​CAG​GGG​CT-3′), *Ctsk* (Forward: 5′-GGGAGAAAAACCTGA AGC-3’; Reverse: 5′-ATT​CTG​GGG​ACT​CAG​AGC-3′), *c-Fos* (Forward: 5′-GCG​AGC​AAC​TGA​GAA​GAC-3′; Reverse: 5′- TTG​AAA​CCC​GAG​AAC​ATC- 3′), and *Hprt* (Forward: 5′-CAG​TCC​CAG​CGT​CGT​GAT​TA-3′’; Reverse: 5′-TGG​CCT​CCC​ATC​TCC​TTC​AT-3′) was used as a housekeeping gene. The perforation of the reaction was running on the ViiA^™^ 7 Real-time PCR machine (Applied Biosystems, Paisley, United Kingdom).

### Luciferase Reporter Assays

The luciferase reporter construct was performed to investigate the activation of NFATc1 and NF-κB transcription with the transfected RAW264.7 cells (Wang et al., 2003; Cheng et al., 2018). Briefly, the prepared transfected cells were seeded in the 48-well plate with 1.0 × 10^5^ cells per well overnight. After the pretreatment with 50 μM RH for 1 hour, the cells were cultured with the presence of RANKL (100 ng/ml) about 24 hours for NFATc1 luciferase reporter and 6 hours for NF-κB luciferase reporter. The cells were subjected to luciferase reporter assay system according to the manufacturer’s protocol (Promega).

### Western Blot Analysis

The BMMs were cultured in 6-well plates and with the adding of RANKL (50 ng/ml) at the concentration of 1.5×10^5^ per well, with the presence or absence of RH in the medium. At the stated culturing times, the cells were lysed in special RIPA lysis buffer. After the cells protein samples were resolved and collected, the SDS gel electrophoresis was used for protein separation. The protein was then transferred to Nitrocellulose blotting membranes (Amersham, Germany). The membranes were gently blocked with 5% skim milk for 2 hours, and incubated in the targeted specific primary antibodies by gently shaking overnight at 4°C. The following day, with the wash by PBS for 3 times per 10 minutes, the membranes were incubated in the secondary Rat/Mouse antibodies for 1 hour at the room temperature. Finally, the antibody reactivity was detected with Extreme Sensitivity Chemiluminescence Substrate (PerkinElmer, Waltham, MA, Unites States). The protein images were visualized on the machine of Image Quant LAS 4000 (GE Healthcare) and then analyzed with the tool of ImageJ software.

### Intracellular Ca^2+^ Measurement

Intracellular Ca^2+^ oscillation assay was used to measure the effects of RH on the calcium signalling, involving in the progress of the osteoclast differentiation as previously described (Wang et al., 2019). Briefly, after the culturing of BMMs for overnight in 48-well plates, the cells were pretreated 1 hour with or without RH (50 μM) and then added RANKL to the complete medium for another coculturing of 24 hours. The treated cells were washed with assay buffer, consisting of Hanks’ buffer and the supplement of 1 mmol/L probenecid and 1% Fetal Bovine Serum. Then the intracellular free calcium was labeled by Fluo4 staining solution for 45 minutes (Chen et al., 2019a). Then the free calcium was detected at the fluorescent light (488 nm excitation wavelength) and the images were scanned and obtained at 2s intervals for 3 minutes with the fluorescence microscope. Oscillating cells with two intensity peaks at the observed time were positively identified and their oscillation intensity changes were counted by the minus of the maximum and the minimum peak (Wang, et al., 2019).

### Statistical Analysis

All data were presented as mean ± standard deviation, representing at least three experiments and performance in triplicate. One-way analysis of variance and Student’s t-test was used to determine the significance of differences between results, with *p* < 0.05 considered to be significant.

## Results

### RH Inhibits RANKL-Induced Osteoclastogenesis

**FIGURE 1:**
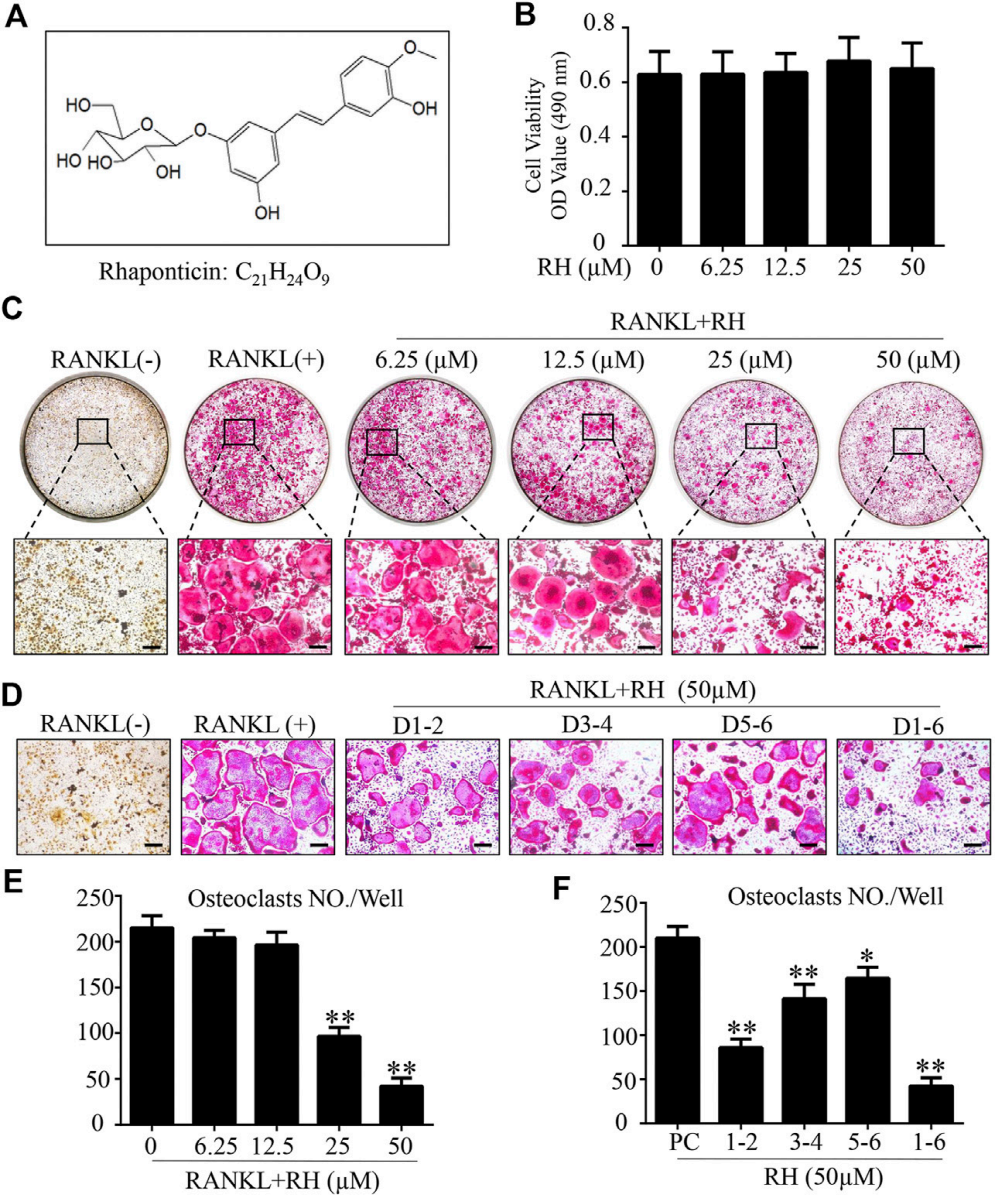
Rhaponticin (RH) suppressed RANKL-induced osteoclastogenesis *in vitro*. **(A)** Chemical structure and formula of RH. **(B)** MTS assay of the BMMs cultured with various concentrations of RH. **(C, D)** Representative images of optical microscope and TRAcP staining of BMMs treated with RH in different concentrations for 6 days **(C)** and in 50 μM, at different time phase of 1–2 days (D1-2), 3–4 days (D3-4), 5–6 days (D5-6), 1–6 days (D1-6), during differentiation **(D)** were shown. **(E, F)** Quantification of TRAcP positive multinucleated osteoclasts (nuclei > 3) with the treatment of RH (*n* = 3). *p < 0.05, **p < 0.01 relative to RANKL-induced control group. Scale bar = 200 μm.

**FIGURE 2:**
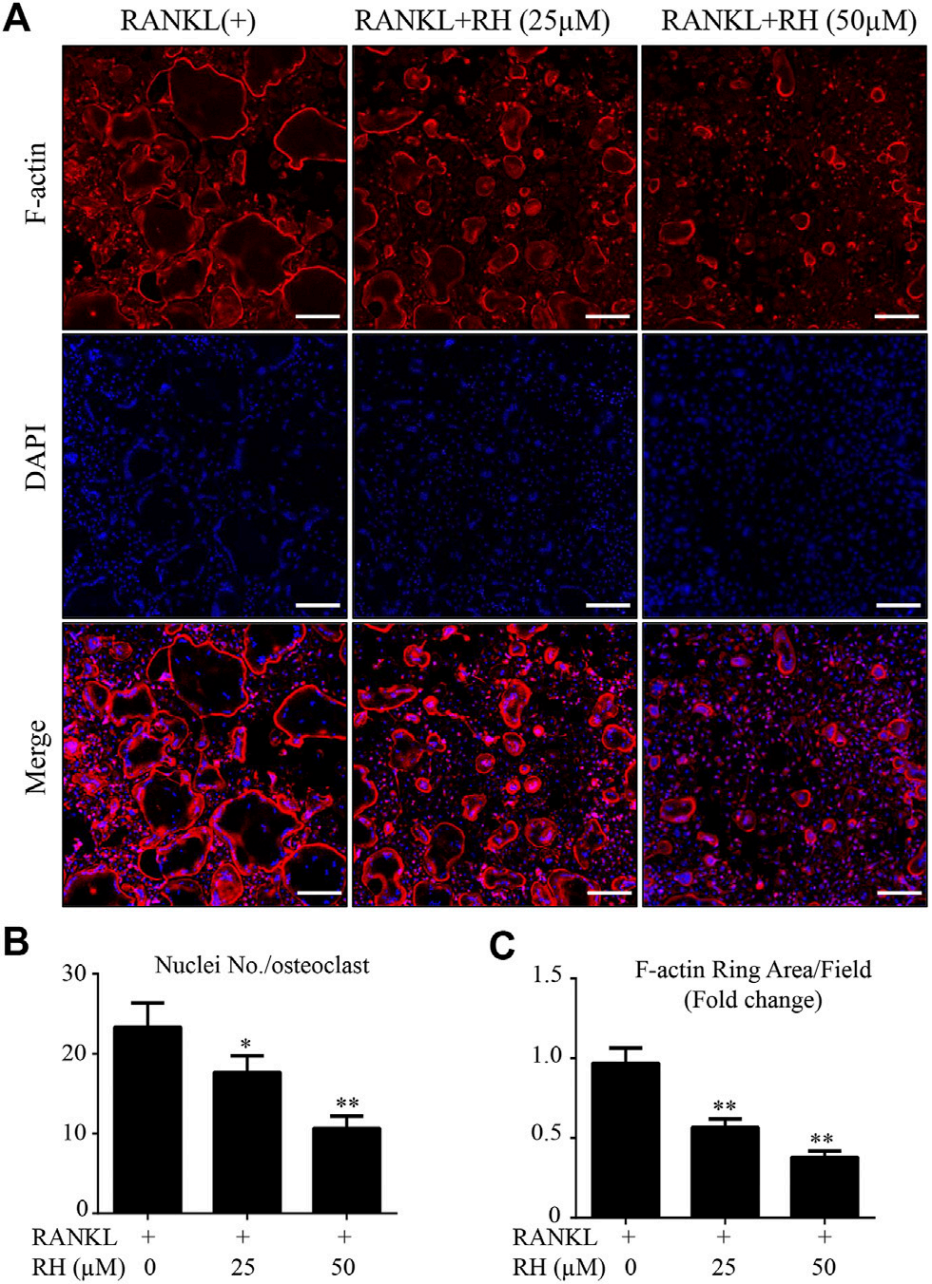
RH inhibited the formation of F-actin belt during the osteoclast formation induced by RANKL. **(A)** The confocal images of F-actin ring formation were detected with Rhodamine Phalloidin, combined DAPI staining for nuclei respectively. Scale bar, 200 μm. **(B)** Quantification of the nucleus number per osteoclast under the different concentrations of RH. **(C)** Quantification of the average of actin ring area per field (*n* = 3). *p < 0.05, **p < 0.01 relative to RANKL-induced control group. Scale bar = 200 μm.

The MTS assay was performed to assess the cytotoxicity of RH on BMM cells. BMMs were cultured with M-CSF and RH for 48 hours at the varying dosages. RH had no effect on the proliferation and viability of BMM cells when compared with the control group at the concentration of 50 µM or lower (Figure 1A and 1B). To determine the influence of RH on the formation progress of osteoclasts from BMMs induced by RANKL, we performed an osteoclastogenesis assay as mentioned above (Chen et al., 2019). The primary BMMs were cultured with the presence of M-CSF and the stimulation of RANKL around 6 days by the treatment of RH in various concentrations. The TRAcP staining result indicated that RH could significantly reduce the osteoclastogenesis in a dose-dependent manner. The TRAcP-positive multinucleated cell numbers at 25 and 50 µM concentrations were significantly less than the positive control group (Figures 1C,E). In the time-course experiment, BMM cells were treated with RH at different time phases (1–2, 3–4, 5–6, 1–6 days), which could investigate the stage that the inhibitory effects of RH treatment mainly exhibits. The results suggested that RH displayed the various levels of effect at the different stages during osteoclast differentiation (Figures 1D,F). To observe the effects of RH on the morphological changes of F-actin ring, a vital structure during the formation of mature OC, BMMs were stimulated with RANKL and the treatmnent of doses of RH until they became mature osteoclasts, then the Rhodamine Phalloidin was used to stain actin ring and DAPI for the nuclei. The nuclei number of the osteoclasts was counted under the microscope (Figure 2A). It showed that both nuclei number per osteoclast and average area of F-actin belt per field were decreased in the presence of RH as compared with that in the RANKL-positive group (Figures 2B,C). Therefore, RH may possess potential inhibitory effects on the osteoclastogenesis induced by RANKL.

### RH Decreases the Hydroxyapatite Resorption by Osteoclastic Cells

**FIGURE 3:**
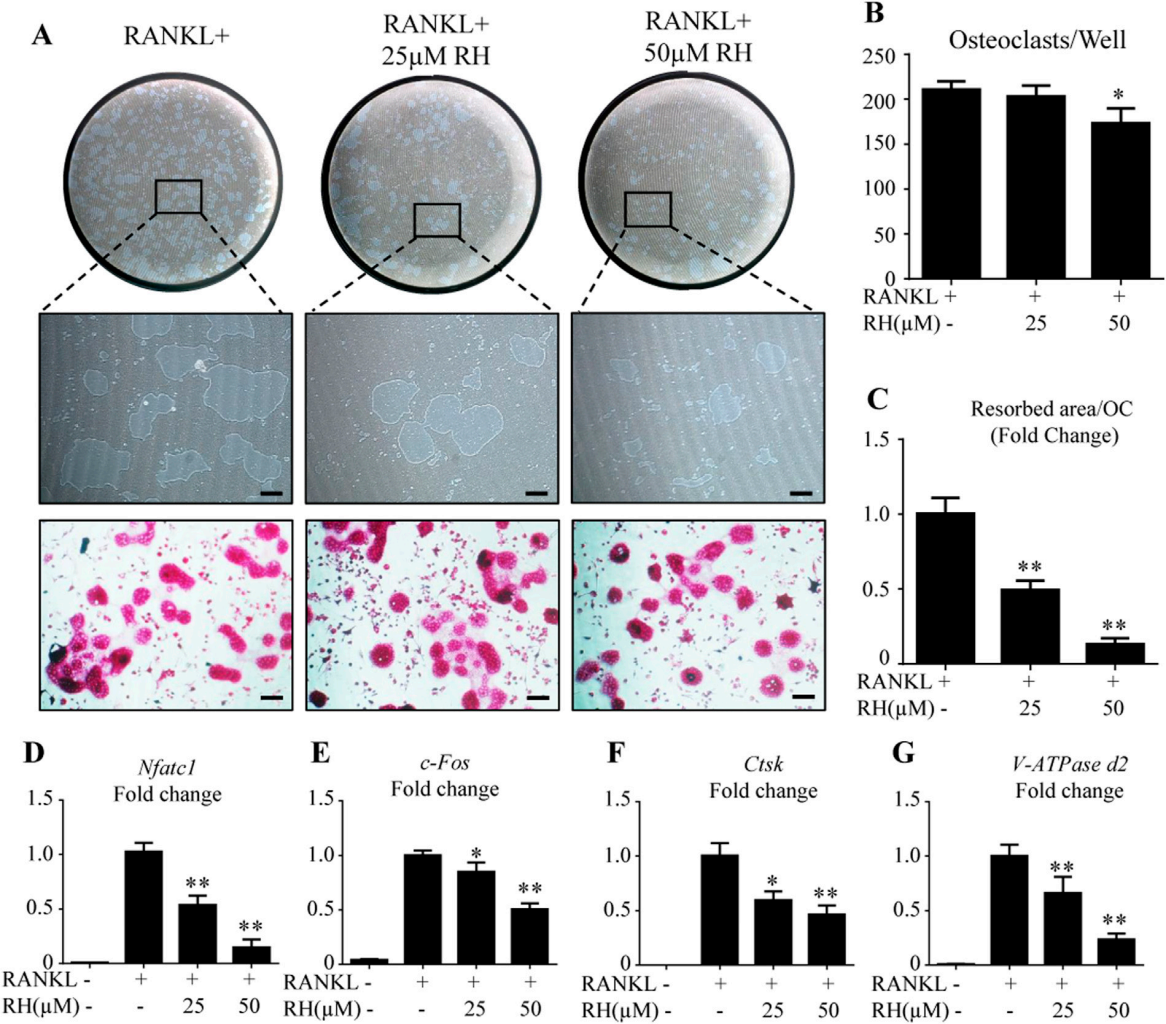
RH attenuated osteoclast hydroxyapatite resorption and osteoclast-specific genes expression. **(A)** Representative images of the resorption on hydroxyapatite-coated plates and TRAcP staining after treatment of RH for 48 hr. **(B)** Quantification of TRAcP-positive osteoclasts numbers per well (n = 3). **(C)** Quantification of resorption area on hydroxyapatite surface per osteoclast (n = 3). **(D–G)** PCR results of osteoclast-specific genes *Nfatc1, c-Fos, Ctsk*, and *Atp6v0d2*. Gene expression levels were standardized to Hprt expression. *p < 0.05, **p < 0.01 relative to RANKL-induced control group. Scale bar = 200 μm.

As RH could inhibit the osteoclastogenesis and formation of F-actin ring, hydroxyapatite resorption assay was carried out to examine the outcome of RH on osteoclast-induced bone resorptive function. Following the incubation of mature osteoclasts with RANKL for 48 hours, the number of osteoclasts was reduced by RH at 50 µM (Figures 3A,B), and the average resorbed area in hydroxyapatite-coated well by per cell was significantly decreased by the treatment of RH (25 and 50 µM) in comparison with the control group (Figure 3C). The hydroxyapatite resorption result was consistent with osteoclastogenesis assay, demonstrating that RH possesses potential anticatabolic influence on osteoclast formation and bone resorptive function.

### RH Inhibits Osteoclast Marker Gene Expression

To further explore the mechanisms of inhibitory effects on osteoclastogenesis and osteoclastic bone resorption by RH, BMMs were cultured with RANKL and M-CSF and treated with RH (25 and 50 µM) for about 6 days till the mature osteoclasts formed. PCR assay was then performed to distinguish the expression levels of osteoclast cells-related marker genes including *Nfatc1, c-Fos, Ctsk*, and *Atp6v0d2*. As demonstrated in Figure 3, the expression of *Nfatc1*, was effectively inhibited by RH in a dose-dependent manner when compared with the control group (Figure 3D). Additionally, the expression levels of other genes, such as *c-Fos*, *Ctsk*, and *Atp6v0d2* were also down-regulated by RH treatment (Figures 3E–G).

### RH Suppresses the Expression Activity of NFATc1 and the Related Proteins

**FIGURE 4:**
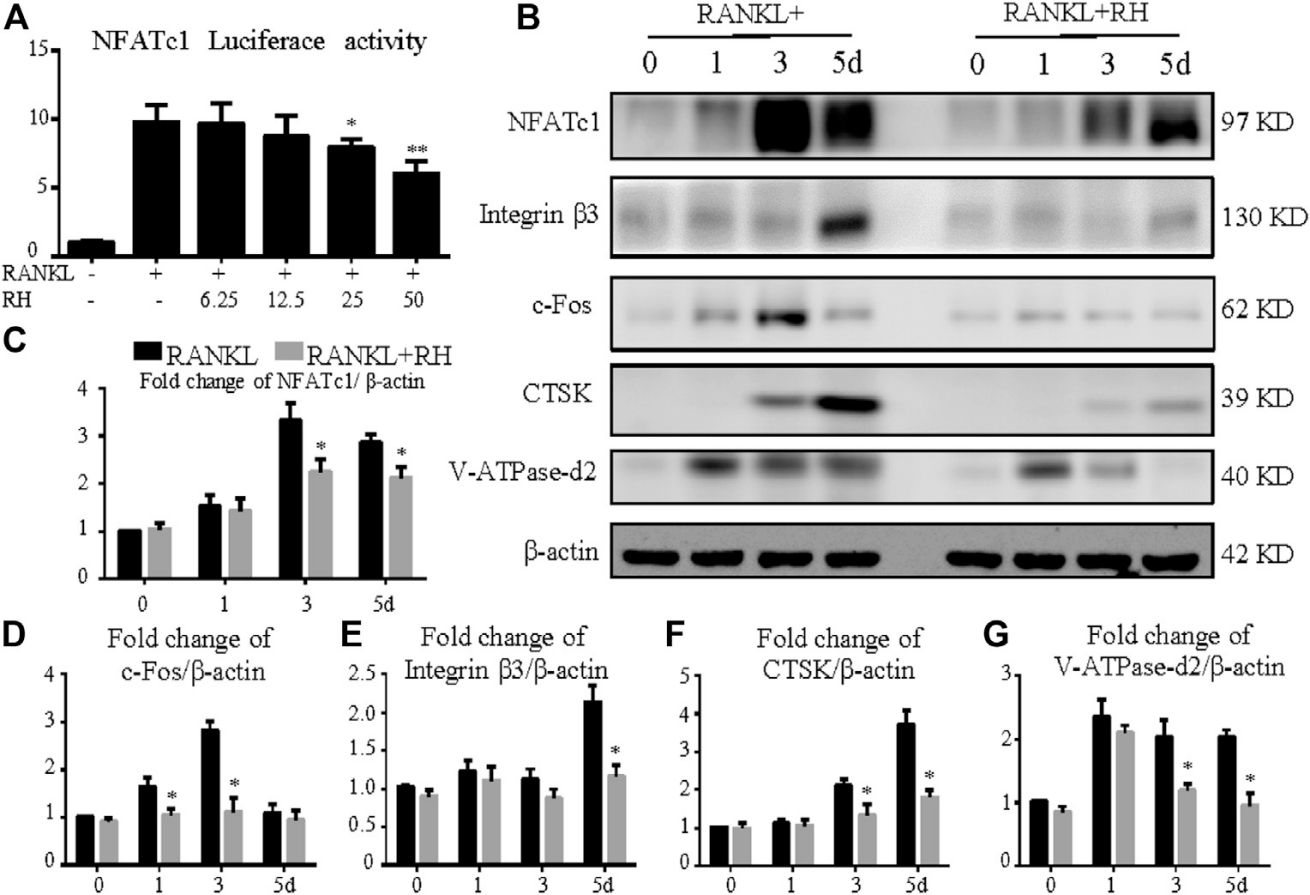
RH suppressed NFATc1 activation and its downstream protein expression. **(A)** RH inhibited the NFATc1 activity of RAW 264.7 cells transfected with luciferase report construct. The cells were pretreated with RH for 1 hour, followed by RANKL stimulation at 50 ng/ml concentration for 24 hours (*n* = 4). **(B)** Representative Western blot images of the effects of RH on the protein expression of NFATc1, integrin β3, CTSK, c-Fos, and V-ATPase-d2 at day 0, 1, 3, and 5 with the stimulation of RANKL (50 ng/mL). **(C–G)** Quantification of the ratios of band intensity of NFATc1, integrin β3, CTSK, c-Fos, and V-ATPase-d2 relative to *β*-actin (*n* = 3). *p < 0.05, **p < 0.01 relative to RANKL-induced control group.

To determine the effects of RH on NFATc1 transcriptional activity induced by RANKL, the NFATc1 luciferase assay was performed. RH pretreatment showed a significant inhibition on RANKL-induced NFATc1 activity (Figure 4A). Additionally, the Western blot results showed that RH could significantly suppress the NFATc1 expression at protein level in osteoclast cells following the induction of RANKL at day 3 and 5 (Figures 4B,C). And the other expressions of osteoclast-related proteins were also down-regulated with RH, including integrin β3, c-Fos, CTSK, and V-ATPase-d2 when compared with the untreated group (Figure 4B-G). Thus, the NFATc1 activity was significantly inhibited via influencing downstream signaling and transcription by RH.

### RH Suppresses ROS Activity and Ca^2+^ Oscillation in the Osteoclastogenesis Induced by RANKL

**FIGURE 5:**
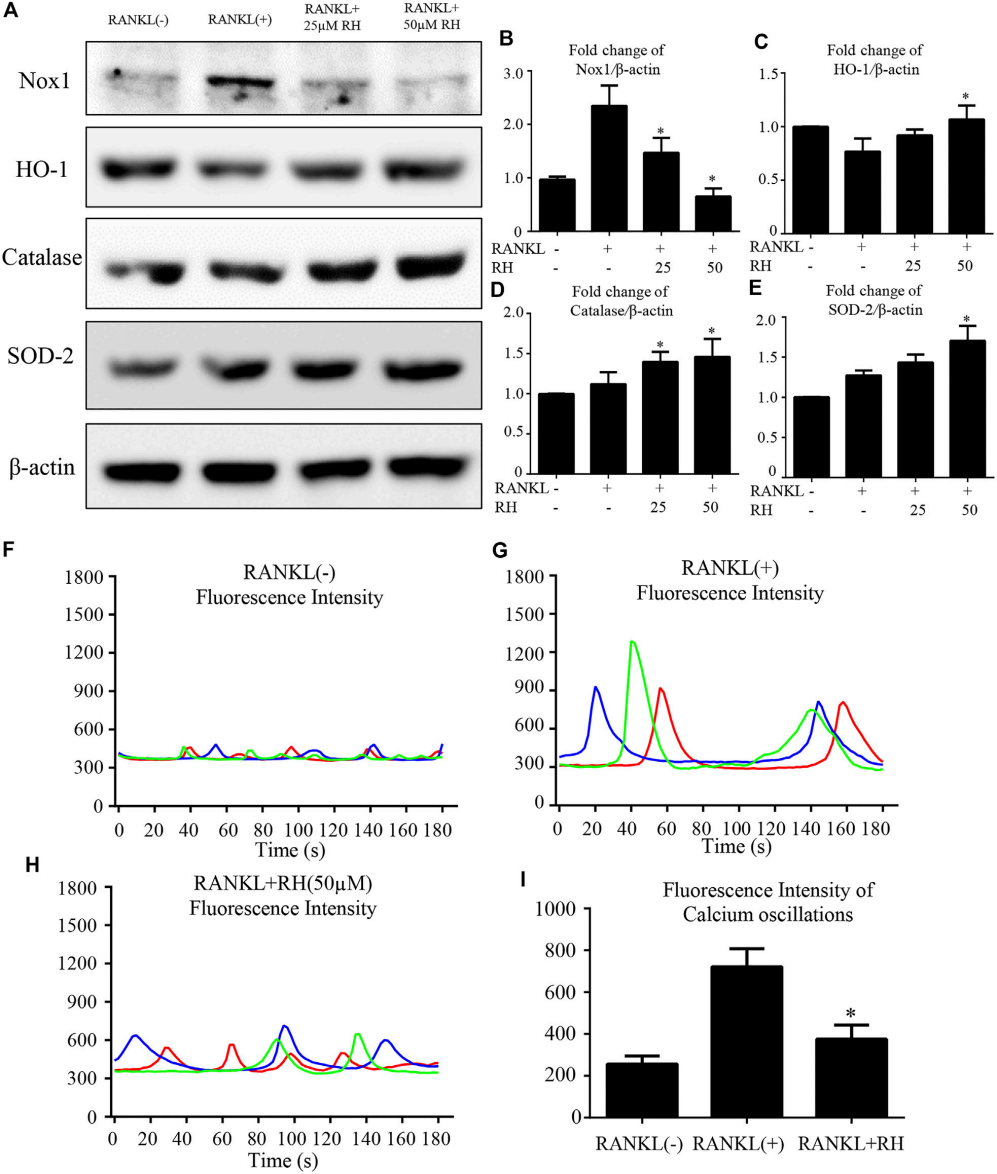
RH suppressed RANKL-induced ROS activity and Ca^2+^ oscillation. **(A)** Representative Western blot images of the Nox1, catalase, HO-1, and SOD-2 expression with the treatment of RH, the Nox1 expression was significantly suppressed by RH, and the antioxidant enzymes of HO-1, catalase and SOD-2 were enhanced. BMMs were stimulated with RANKL (50 ng/mL) with RH at the concentration of 25 and 50 μM or PBS for 2 days before collecting protein. **(B–E)** Quantification of the ratios of band intensity of Nox1, HO-1, catalase, and SOD-2 relative to *β*-actin (*n* = 3 per group). *p < 0.05, **p < 0.01 comparison with the RANKL-induced positive control group. **(F–H)** Representative images of fluorescence intensity waves of Ca^2+^ oscillation in negative group, RANKL stimulated positive group and RH (50 μM) treated group. There were three colours indicating different cells in each group. **(I)** Quantification of fluorescence intensity change of Ca^2+^ oscillation in each group. *p < 0.05, **p < 0.01 relative to RANKL-induced positive control group.

To inspect the effect of RH on the intracellular ROS level at the stage of osteoclast differentiation induced by RANKL, oxidation-sensitive protein makers were tested with Western blot assay. Nox1 was known as the protein marker contributing to ROS generation (Sasaki et al., 2009). The Nox1 protein expression level was significantly improved by the stimulation of RANKL, but significantly suppressed with the treatment of RH (Figures 5A,B). The antioxidant enzymes including HO-1, catalase and SOD-2 were enhanced dose-dependently by RH treatment as examined by Western blot (Figures 5A,C–E). The results indicated that RH is able to reduce the intracellular ROS generation and enhance the scavenging ability of ROS level. As reported by other studies, calcium oscillation initiation plays a grave role in the activation of NFATc1 (Takayanagi et al., 2002). Furthermore, to explore the molecular mechanism of the inhibition of osteoclast formation and function by RH, the intracellular calcium oscillation activity was also performed. In the results, Ca^2+^ oscillation was increased with the stimulation of RANKL. While this trend was dramatically attenuated after the treatment of RH (Figures 5F–I).

### RH Represses NF-κB Activation and the Phosphorylation of ERK and P38 in MAPK Pathways

**FIGURE 6:**
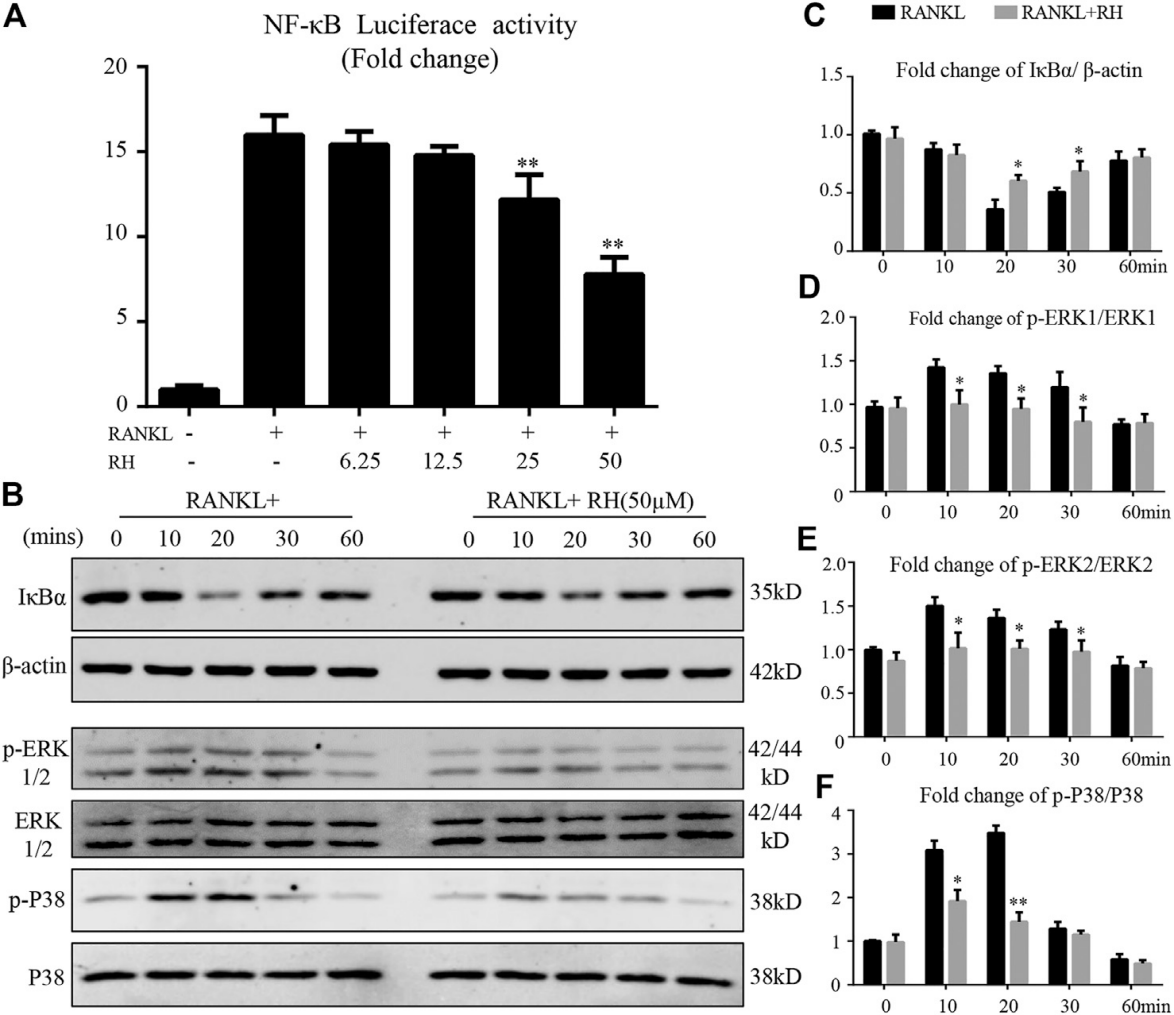
RH suppressed the activation of NF-κB and the phosphorylation of ERK and P38 induced by RANKL. **(A)** RH inhibited the NF-κB activity of RAW 264.7 cells with luciferase report construct at the different concentrations of RH as indicated. The cells were pretreated with varying densities of RH and stimulated with RANKL at 50 ng/mL for 6 hours. **(B–C)** Representative images of the expression level of IĸB-α and *β*-actin in Western blot assay and the quantification of the ratios of band intensity of IκB-α to *β*-actin. **(B–F)** Representative images of the expression phosphorylation level of ERK and P38 with or without the treatment of 50 μM RH, and the quantification of the fold change ratios of band intensity of p-ERK to ERK and p-P38 to P38 (*n* = 3). *p < 0.05, **p < 0.01 relative to RANKL-induced positive group.

We also investigated the effects of RH on NF-κB activity induced by RANKL. With the luciferase reporter assay, RANKL stimulation could increase the activity of NF-κB luciferase, while the treatment of RH significantly inhibited the obvious trend (Figure 6A). NF-κB complex is bound with IκB-α and is prevented from translocating to nucleus for futher activation. Western blot results further indicated that the degradation of IκB-α was induced by RANKL but was suppressed by RH treatment (Figures 6B,C). Therefore, it was revealed that RH treatment had an attenuating effect on NF-κB activity during the progress of osteoclastogenesis.

The MAPK signalling pathway members of P38 and ERK are vital in the activation of c-Fos and NFATc1 (Chen et al., 2019a; Huang, Chang, et al., 2006). Therefore, the effects of RH treatment on MAPK pathway activation during RANKL-induced osteoclastogenesis was investigated. BMMs were preincubated in serum-free medium for 4 hours, then stimulated by RANKL with the presence of RH or not, at 0, 10, 20, 30, and 60 minutes. RH pretreatment had attenuated the phosphorylation of ERK at 10, 20, and 30 minutes (Figures 6B,D,E). Additionally, the phosphorylation of P38 was significantly inhibited by RH treatment at 10 and 20 minutes compared to the control group (Figures 6B,F). These results suggested that RH could down-regulate osteoclast formation and function against the RANKL stimulation by suppressing the MAPK signalling pathways.

## Discussion

Osteoporosis is a highly prevalent disease which can cause a substantial economic burden to individuals and society due to the increased risk of bone fractures (Harvey, Dennison, & Cooper, 2010). And the excessive osteoclastic activity and bone resorption are considered as the main reasons for bone loss (Baron & Kneissel, 2013). The serious side effects induced by the long-term and large-usage of the traditional clinical anti-osteoporotic agents have limited their wide application in treating osteoporosis (Lopez-Jornet, et al., 2010; Ross, et al., 2000; Wang, et al., 2017). Therefore, alternative therapeutic agents to prevent osteolytic diseases are urgent to be exploited. Many herbs have been proved possessing the effects to treat osteoporosis (Chen et al., 2019; Jin et al., 2019). RH, an important stilbene-type component extracted from the root of *Rheum undulatum L.*, possesses various biological activities, such as anti-allergic, anti-cancer, anti-diabetic and anti-inflammatory activities in the previous reports (A. Kim & Ma, 2018; Li, Tian, Wang, & Ma, 2014; Tao, et al., 2017). However, there is no research regarding its potential effects on bone cells. In this study, firstly, RH was proved to be able to suppress the osteoclastogenesis and osteoclastic resorption.

Osteoclasts are the monocyte-macrophage lineage-derived large multinucleated cells, acting with the vital role in bone remodelling by resorbing bone matrix. M-CSF and RANKL play a leading role in osteoclast biology and are used to induce osteoclastogenesis in our study. M-CSF provides signals required for the survival and proliferation of early osteoclast precursors. It is also imperative for macrophage maturation in the presence of RANKL. Upon these stimulations, the precursors differentiate into the mature osteoclasts characterized by the F-actin ring forammtion with resorptive function (Teitelbaum, 2000). TRAcP is an acid phosphatase and abundant in osteoclasts, a specific marker for osteoclast cells activity (Janckila, Takahashi, Sun, & Yam, 2001). In our study, the MTS results found that the viability of BMM cells was not influenced by RH, even at the high concentration of 50 μM, while the quantity of RANKL-induced TRAcP-positive cells was meaningfully lower by RH treatment compared with the positive group, indicating the osteoclast formation was efficiently inhibited by RH. Furthermore, in the time-course assay, the number of TRAcP-positive mature osteoclasts was effectively reduced at different times as indicated, especially with fewer osteoclasts at the early and middle stages. It may be related with the early inhibition of RH on the upstream of ROS, NF-κB, and MAPK pathways and then the NFATc1 protein expression according to the results below. Additionally, the mature osteoclasts are featured by resorbing the bone tissues through multiple processes (Croucher, McDonald, & Martin, 2016). In the bone resorption pit assay, we found that RH-treated group displayed a significantly reduced resorption area as compared with the positive control group, indicating the inhibition of RH on bone resorption.

Next, we explored the mechanisms for the anti-catabolic effects of RH. During osteoclast differentiation and maturation stimulated by RANKL, the BMM will highly express NFATc1 protein at about 2 or 3 days after the stiulation of RANKL, acting as a master transcriptional factor to promote the expression of the numerous genes which are prerequisites for bone resorption (Negishi-Koga & Takayanagi, 2009; Zhao, Wang, Liu, He, & Jia, 2010). The physiological implication of NFATc1 for osteoclasts has been well elucidated. Firstly, osteoclast precursor cells with the deficiency of NFATc1 fail to differentiate into the mature osteoclasts (Zhao, Shao, Chen, & Li, 2007). Secondly, NFATc1 regulates the expression of target genes and proteins that empower osteoclastic resorption as evidenced by the osteopetrosis in the conditional NFATc1-deficient mice (Aliprantis et al., 2008). In our study, we found that RH significantly suppressed the expressions of NFATc1 at both gene and protein levels, which further caused the downregulation of related proteins and genes including integrin β3, c-Fos, V-ATPase-d2, and CTSK. This was also supported by the suppressed NFATc1 transcriptional activity as examined by luciferase assay. These findings suggested the involvement of NFATc1-mediated mechanisms by which RH inhibited osteoclastogenesis and osteoclastic resorption.

The physiological intracellular ROS activity rests on the equilibrium between the rates of generation and scavenging (Chen et al., 2019). RANKL-induced ROS production is mainly mediated by Nox1 (Lee et al., 2005) and ROS were essential in mediating osteoclastogenesis by facilitating MAPKs and NF-κB (Koh et al., 2006; Kim, Lee, Kim, Lee, & Kim, 2017). Our results showed that the expression of Nox1 was effectively inhibited by RH. We then examined the RH’s effect on antioxidant enzymes (HO-1, catalase, and SOD-2) which contribute to ROS scavenging. Previous studies showed that HO-1 was an antioxidant protein which can protect organs from the onset of tumorigenesis, and regulate osteoclastogenesis and bone resorption (Zwerina et al., 2005; Chen et al., 2016). Catalase could protect tumour cells against specific apoptosis induction by inhibiting the intercellular ROS generation (Bechtel & Bauer, 2009). SOD-2 was a vital factor for bone metabolism. The knockdown of SOD-2 would be inductive for ROS generation, leading to increasing osteoclasts formation. Whilst SOD-2 overexpression significantly suppressed the differentiation of mature osteoclast by attenuating ROS level (Kim, et al., 2017). These ROS scavenging proteins were significantly upregulated following RH treatment. Taken together, RH suppressed the ROS level during the osteoclastogenesis by significantly inhibiting ROS production and boosting ROS scavenging activity.

Upon RANKL binding to RANK, a series of downstream signalling cascades are initiated to regulate osteoclast formation. The expression of NFATc1 would be activated by the upstream cascades of TNF receptor-associated factor 6 (TRAF6), NF-κB, MAPK, and calcium-signalling pathways (Huang, Ryu, et al., 2006). IκB-α binds to NF-κB complex and prevents its translocation into the nucleus (Ghosh & Hayden, 2008). Following the stimulation of RANKL, IκB-α is degraded in the cytoplasm and releases NF-κB which subsequently induces osteoclastogenesis. Our results demonstrated that the treatment of RH inhibits the degradation of IκB-α in the presence of RANKL, indicating the suppression on NF-κB activity.

Furthermore, ERK and P38 are the essential members of the MAPK family. RANKL could enhance the phosphorylation levels of ERK and P38 to regulate the differentiation and function of osteoclasts (Liu et al., 2019). It was reported that the inhibitors of P38 (SB202190) and ERK (PD98059) could block the activation of the MAPK pathway and thus exerted a strong suppression on RANKL-induced osteoclast formation (Lee et al., 2002). The Western blot results demonstrated that RH could inhibit the expression levels of p-ERK1/2 and p-P38 induced by RANKL. This may also partly due to the decreased ROS level following RH treatment. The longlasting Ca^2+^ oscillation is essential to guarantee the vigorous induction of NFATc1 in an auto-amplification manner, which is initially triggered by the stimulation of RANKL (Asagiri et al., 2005). Calcineurin inhibitors potently suppressed RANKL-induced osteoclast formation via decreasing the translocation of nuclear NFATc1, suggesting the crucial role of the Ca^2+^-NFATc1 pathway in osteoclastogenesis (Takayanagi, et al., 2002). Interestingly, we found that RH treatment downregulated the activity of RANKL-induced Ca^2+^ oscillation, which may largely contribute to the suppression of NFATc1 in our study.

**FIGURE 7:**
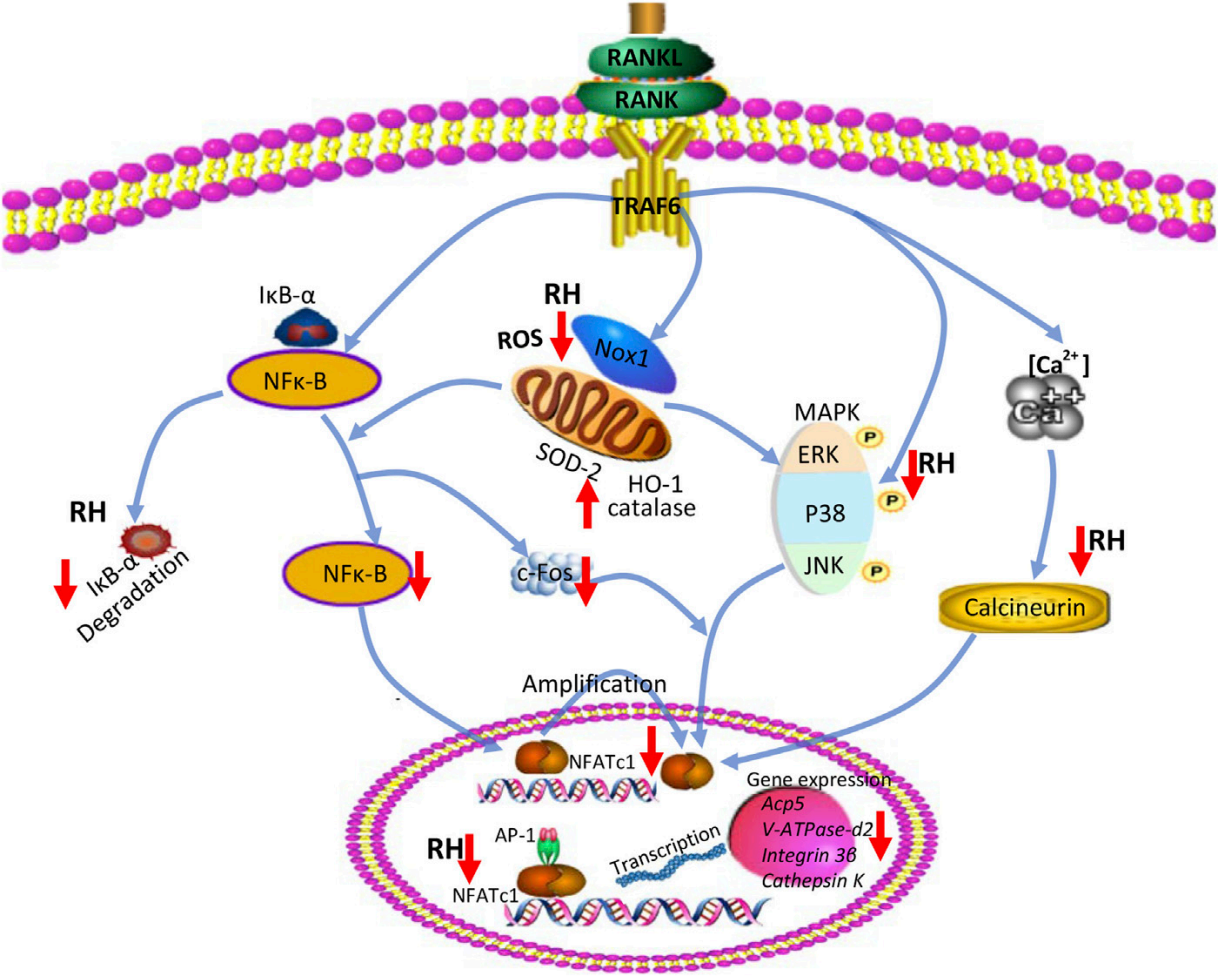
A proposed diagram depicts the RH treatment on the inhibition of osteoclast formation and function. RH suppresses NF-κB activity, ERK, and P38 phosphorylation and Ca^2+^ oscillation, eventually leading to the deactivation of NFATc1 and its downstream osteoclast-specific genes. In addition, RH treatment also reduced ROS level by inhibiting ROS production and boosting ROS scavenging activity.

In summary, our studies indicated that RH has inhibitory effects on RANKL-induced osteoclastogenesis and osteoclastic resorption via effecting vital signaling including ROS, NF-κB, MAPK, Ca^2+^ oscillation, and eventually NFATc1 activation (Figure 7). The results propose that RH, a natural compound from *Rheum undulatum L.*, may be a potential and therapeutic candidate for the prevention and treatment of osteoclast-related bone disorders. However, in our study, the effected concentration for RH to exhibit the antiosteoporotic function is about 50μM, without cellular toxicity for BMMs, so the higher concentration should be considered in the future studies. Meanwhile the therapeutic effects on osteoporosis *in vivo* and the precise molecular target of RH in osteoclasts may require further investigations.

## Data Availability

The original contributions presented in the study are included in the article/Supplementary material, further inquiries can be directed to the corresponding authors.
