# Corrigendum: Inhibitory effects of Rhaponticin on osteoclast formation and resorption by targeting RANKL-induced NFATc1 and ROS activity

**DOI:** 10.3389/fphar.2023.1297863

**Published:** 2023-12-18

**Authors:** Jianbo He, Kai Chen, Tiancheng Deng, Jiewei Xie, Kunjing Zhong, Jinbo Yuan, Ziyi Wang, Zhifeng Xiao, Ronghe Gu, Delong Chen, Xiaojuan Li, Dingkun Lin, Jiake Xu

**Affiliations:** ^1^ The Second Affiliated Hospital of Guangzhou University of Chinese Medicine, Guangdong Provincial Hospital of Chinese Medicin, Zhuhai, China; ^2^ School of Biomedical Sciences, University of Western Australia, Perth, WA, Australia; ^3^ Department of Orthopedics, First People’s Hospital of Nanning, Fifth Affiliated Hospital of Guangxi Medical University, Nanning, China; ^4^ The First Affiliated Hospital of Guangzhou University of Chinese Medicine, Guangzhou, China; ^5^ Formula-Pattern Research Center, School of Traditional Chinese Medicine, Jinan University, Guangzhou, China

**Keywords:** osteoclast, NFATc1, ROS, rhaponticin, bone

In the published article, there was an error in Figure 3A about the 50 μM TRAcP picture as published. Two pictures were taken from the same cell culture well with 25 μM treatment, and partially overlapped images were obtained during photo taking, because both were from the same well. However, one image was placed as a 50 μM group, which occurred during the process of PowerPoint figure preparation due to an oversight. The corrected Figure 3 appears below.

**FIGURE 3:**
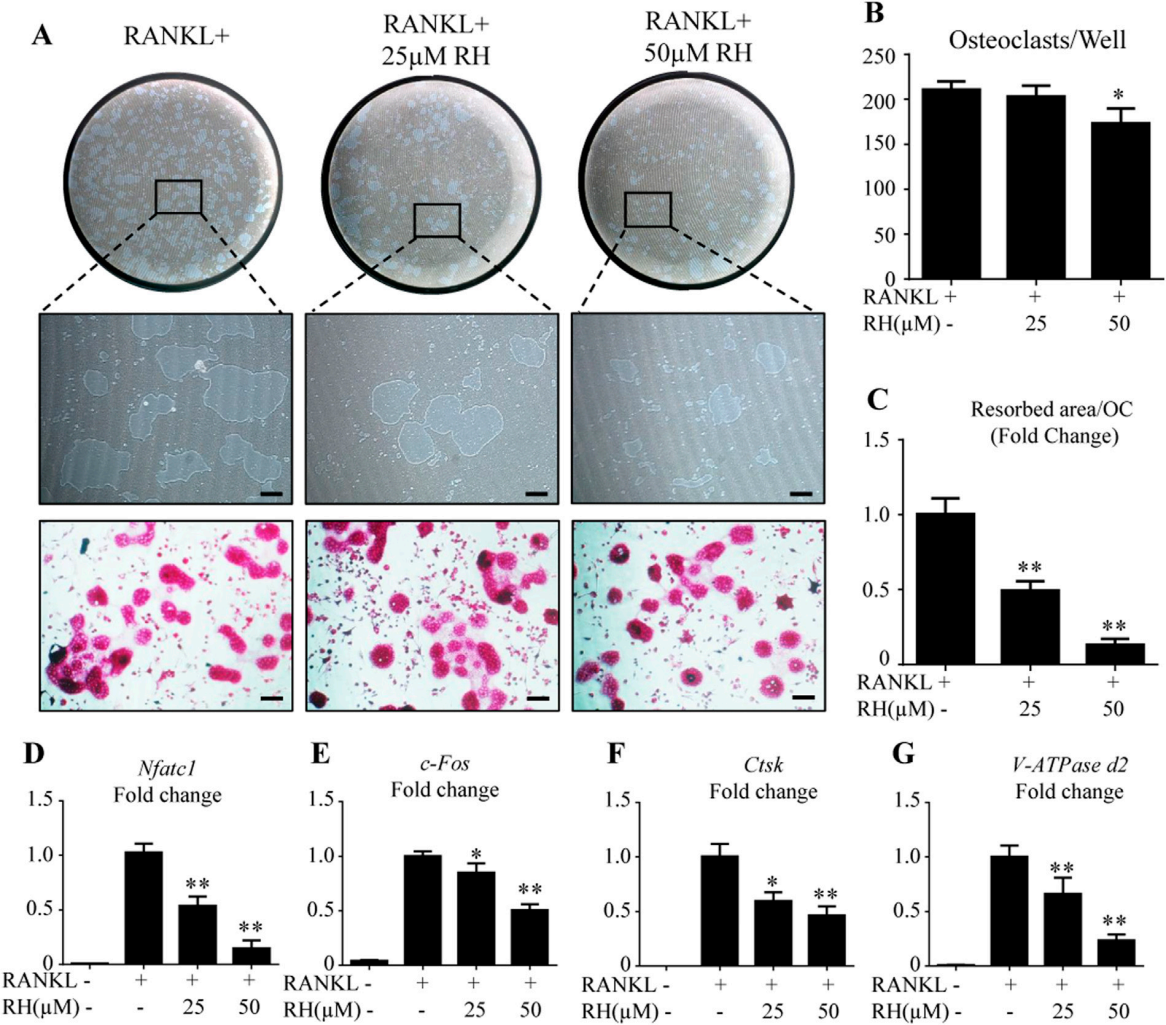
RH attenuated osteoclast hydroxyapatite resorption and osteoclast-specific genes expression. **(A)** Representative images of the resorption on hydroxyapatite-coated plates and TRAcP staining after treatment of RH for 48 hr. **(B)** Quantification of TRAcP-positive osteoclasts numbers per well (n = 3). **(C)** Quantification of resorption area on hydroxyapatite surface per osteoclast (n = 3). **(D–G)** PCR results of osteoclast-specific genes *Nfatc1*, *c-Fos*, *Ctsk*, and *Atp6v0d2*. Gene expression levels were standardized to Hprt expression. *p < 0.05, **p < 0.01 relative to RANKL-induced control group. Scale bar = 200 μm.

The authors apologize for this error and state that this does not change the scientific conclusions of the article in any way. The original article has been updated.

